# Perpendicular Vascular Changes in NBI-CE of Laryngeal Lesions: Diagnostic Accuracy, Reproducibility, and Common Pitfalls

**DOI:** 10.3390/diagnostics15233051

**Published:** 2025-11-29

**Authors:** Paul Pickert, Anja Giers, Anke Lux, Vassiliki-Anna Papaioannou, Nazila Esmaeili, Jannis Hagenah, Alfredo Illanes, Axel Boese, Christoph Arens, Nikolaos Davaris

**Affiliations:** 1 Department of Otorhinolaryngology, Head and Neck Surgery, Otto-von-Guericke University Magdeburg, 39106 Magdeburg, Germany; paul.pickert@uk-augsburg.de (P.P.);; 2 Department of Ophthalmology, University Hospital Augsburg, 86156 Augsburg, Germany; 3 Institute of Biometry and Medical Informatics, Otto-von-Guericke University Magdeburg, 39106 Magdeburg, Germany; 4 Department of Otorhinolaryngology, Head and Neck Surgery, Charité—Universitätsmedizin Berlin, 10117 Berlin, Germany; 5 Center for Digital Surgery, Department of General, Visceral and Pediatric Surgery, University Medical Center Göttingen, 37073 Göttingen, Germany; 6 Fraunhofer Research Institution for Individualized and Cell-Based Medical Engineering (IMTE), 23562 Lübeck, Germany; 7 Faculty of Computer Science & Research Campus STIMULATE, Otto-von-Guericke University Magdeburg, 39106 Magdeburg, Germany; 8 INKA, Institute for Intelligent Catheter Applications, Otto-von-Guericke University Magdeburg, 39106 Magdeburg, Germany; 9 Head and Neck Surgery, and Plastic Surgery, Department of Otorhinolaryngology, Justus Liebig University of Giessen, 35392 Giessen, Germany

**Keywords:** diagnostic accuracy, enhanced contact endoscopy (ECE), European Laryngological Society (ELS) classification, interobserver agreement, laryngeal lesions, narrow-band imaging (NBI), perpendicular vascular changes (PVC), pitfalls

## Abstract

**Background/Objectives**: Differentiating benign, premalignant, and early malignant vocal fold lesions is challenging. Perpendicular vascular changes (PVCs) per the European Laryngological Society (ELS) are key malignancy indicators. Enhanced contact endoscopy with narrow-band imaging (NBI-CE) visualizes intrapapillary capillary loops (IPCLs) at high magnification, independent of gross morphology. However, defining malignancy as any PVC increases sensitivity but lowers specificity—particularly in papillomas—whereas limiting malignancy to narrow-angle PVC improves specificity but risks false negatives and reduced reproducibility. **Methods**: We intraoperatively evaluated 146 histology-proven vocal fold lesions using NBI-CE. Six raters (three experienced otolaryngologists, three PhD students) classified vascular patterns. Two approaches were tested: (1) malignancy = narrow-angle PVC; (2) malignancy = any PVC. Outcomes were accuracy, sensitivity, specificity, and interrater agreement. **Results**: Approach (1) had higher specificity but lower sensitivity than (2) (~85% vs. ~70% specificity; ~50% vs. ~80% sensitivity). Accuracy did not differ significantly. Experienced raters showed higher interrater agreement and a more favorable sensitivity–specificity balance. Common errors were false positives in papillomas and false negatives in dysplasia/early carcinoma. **Conclusions**: PVC assessment with NBI-CE is feasible and informative. Choosing between “any PVC” and “narrow-angle only” entails a sensitivity–specificity trade-off and depends on lesion type and experience. Refined ELS descriptors and automated analysis may improve reproducibility and accuracy.

## 1. Introduction

Early recognition of high-grade dysplasia or malignancy in vocal fold lesions remains one of the major diagnostic challenges in laryngology, and vascular changes have emerged as one of the most informative features for detection and differentiation [1,2].

Such vascular alterations can traditionally be detected in white-light (WL) endoscopy, but their visibility is significantly enhanced by image-enhanced endoscopy (IEE) techniques [2,3,4]. Among these, narrow-band imaging (NBI) is the most extensively studied modality, with several investigations demonstrating superior sensitivity compared with WL endoscopy for detecting premalignant and malignant laryngeal lesions [5,6,7]. Beyond improved sensitivity, NBI also enhances observer reliability in overview endoscopy, increasing both inter- and intraobserver agreement compared with white light imaging [8]. More recently, Yildirim et al. demonstrated that IMAGE1 S™ can likewise improve the evaluation of vascular changes in accordance with standardized classification systems [9].

When contact endoscopy (CE) is combined with IEE, the method is referred to as enhanced contact endoscopy (ECE). The most widely studied approach, NBI-CE, enables high-magnification, high-contrast visualization of vascular alterations while largely eliminating the confounding influence of gross lesion morphology. Therefore, ECE is particularly well suited for studying the clinical diagnostic value of vascular changes in isolation [2,10,11,12].

Several classification systems have been proposed to describe vascular changes in laryngeal lesions. Among them, the European Laryngological Society (ELS) classification has gained widespread use because of its simplicity and clinical applicability [2,10,13]. In contrast to more complex grading schemes such as those proposed by Ni or Puxeddu, which subdivide intrapapillary capillary loops into multiple types, the ELS system reduces vascular patterns to a dichotomic distinction between longitudinal vascular changes (LVC), usually associated with benign processes, and perpendicular vascular changes (PVC), which are strongly linked to premalignant and malignant lesions [10,13]. A further subdivision into narrow-angle (naPVC) and wide-angle PVC (waPVC) was suggested to improve the differentiation between papillomas and carcinomas [10,14]. Validation studies in overview NBI have demonstrated that the ELS classification reaches high sensitivity and specificity for distinguishing benign from high-grade and malignant lesions and that it can be applied with good interobserver agreement in the optical biopsy setting [2,4,7,8]. At the same time, comparative work has shown that different vascular classifications do not always assign identical risk categories to the same pattern [2,13].

Despite its broad acceptance, the ELS system still faces limitations, particularly in distinguishing papillomas, dysplasias, and early carcinomas. Papillomas display perpendicular vascular changes, often with wide-angle turning points embedded in exophytic warty structures, thereby mimicking malignant lesions and reducing specificity. Dysplastic lesions, on the other hand, show heterogeneous vascular patterns: high-grade dysplasia and carcinoma in situ frequently exhibit PVC, whereas low-grade dysplasia may present with longitudinal or only subtly altered vessels, which may decrease sensitivity [11,13,14]. Large NBI series have confirmed that perpendicular vascularization is highly prevalent in papillomatosis and malignant lesions but is not entirely absent in benign disease, and that the diagnostic performance of PVC-based assessment varies across histological subgroups and clinical scenarios [3,4].

Moreover, most prior studies in the field relied on overview endoscopy (white light and NBI), where vascular assessment is unavoidably influenced by macroscopic features of the lesion, such as color, keratinization, leukoplakic surface changes, or exophytic growth, rather than by vascular architecture alone. This is particularly relevant in entities such as vocal fold leukoplakia, in which optical impressions of thickness and surface texture strongly shape risk stratification [2,4]. In contrast, enhanced contact endoscopy (ECE/NBI-CE) visualizes the intrapapillary capillary loops at high magnification and allows a more isolated evaluation of vascular morphology, largely independent of gross lesion appearance. However, data on diagnosis-specific misclassification patterns and on reproducibility across different expertise levels when vascular morphology is assessed in isolation with high-magnification NBI-CE remain scarce [11,12,13].

Against this background, the present study aimed to systematically evaluate the diagnostic accuracy and reproducibility of PVC assessment in NBI-CE images of vocal fold lesions. Specifically, we analyzed (1) the diagnostic performance of two PVC-based criteria (any PVC vs. naPVC), (2) the reproducibility among experienced and inexperienced raters, and (3) diagnosis-specific misclassification patterns.

## 2. Materials and Methods

### 2.1. Data Collection

This single-center retrospective study is based on a pre-existing, curated CE-NBI dataset derived from all microlaryngoscopic examinations performed at the Department of Otorhinolaryngology, Head and Neck Surgery, Otto-von-Guericke University Magdeburg, between 1 January 2015, and 31 December 2018. All procedures were carried out by an experienced surgeon in the operating theater under general anesthesia. Indications for surgery included benign, premalignant, or malignant lesions of the vocal folds identified during previous outpatient or inpatient examinations. The data used in this study are part of the published Contact Endoscopy–Narrow Band Imaging (CE-NBI) dataset [15,16]. This openly available dataset was specifically curated for the assessment of laryngeal lesions and has been described in detail in the accompanying data paper.

### 2.2. Intraoperative Setting

Intraoperatively, a 30-degree contact endoscope (Karl Storz, Tuttlingen, Germany) was used, connected to an Evis Exera III video system with a xenon light source and integrated NBI filter (Olympus Medical Systems, Hamburg, Germany). Video sequences of the examinations were recorded. The surgeon was able to switch between conventional white light (WL) endoscopy and the NBI filter at the push of a button. After capturing an overview image of the vocal folds, the lesion of interest and its surrounding mucosa were examined with the contact endoscope in NBI mode, at 60–150× magnification and in direct contact with the mucosa. This enabled detailed visualization of vascular changes. All recordings were stored using RP-Szene software version 10 (Rehder/Partner GmbH, Hamburg, Germany). Subsequently, biopsy, excisional biopsy, or cordectomy was performed for histopathological analysis.

### 2.3. Data Processing

All video sequences were reviewed, and a dataset was created. Still images were pre-selected prior to rating by an investigator not involved in rating, based on focus, illumination, and absence of motion blur. For each patient, three to five representative still images of NBI-CE with the best possible image quality were manually extracted. Care was taken to ensure that the selected images could be clearly assigned to the corresponding lesion. Representative NBI-CE video sequences are provided as Supplementary Material. Video S1 illustrates longitudinal vascular changes (LVC) in a Reinke’s edema, whereas Video S2 demonstrates perpendicular vascular changes (PVC) in a vocal fold papilloma. Analyses were conducted at the patient level; multiple images per patient informed a single decision per rater. Raters were blinded to histopathology, clinical information, and to each other’s ratings. Case order for rating was randomized for each rater to minimize order effects.

### 2.4. Inclusion and Exclusion Criteria

Inclusion criteria comprised adult patients, the availability of at least three high-quality NBI-CE images, and a histologically confirmed diagnosis from the Institute of Pathology according to the WHO classification (WHO 2005 scheme). Cases without definitive histology, with insufficient image quality, or with unclear lesion assignment were excluded during data curation by the authors responsible for constructing the CE-NBI dataset. Overall, fewer than 5% of otherwise eligible cases were excluded due to insufficient image quality.

### 2.5. Classification of Vascular Changes According to ELS Criteria by Independent Raters

All patient cases were evaluated by six raters: three ENT specialists with long-standing experience in laryngology and routine use of NBI-CE in clinical practice (“experienced group”), and three PhD students/junior researchers from the ENT department (“inexperienced group”) with limited clinical experience in laryngology and no routine responsibility for NBI-CE-based decision making. All raters were provided with a training paper giving an overview of the topic. The ELS guideline for the classification of longitudinal vascular changes (LVC) and perpendicular vascular changes (PVC) was presented in both image and text format, along with the distinction between narrow-angle and wide-angle IPCLs, including example images for all histological categories. Raters could assign one of the following categories for each patient case: LVC, narrow-angle PVC, or wide-angle PVC (Table 1). Each case was rated only once per observer in a single rating session; intra-rater reproducibility was not assessed.

### 2.6. Malignancy Criteria and Statistical Analysis

Endoscopic criteria for malignancy in differentiating between benign and malignant lesions were defined as the presence of PVC according to the ELS definition, and alternatively, the presence of narrow-angle PVC (Table 2).

Histological diagnosis served as the gold standard. For the purpose of calculating sensitivity, specificity, and accuracy, mild dysplasias were categorized as benign, while moderate dysplasias, severe dysplasias, and carcinoma in situ (CIS) were categorized as malignant.

Diagnostic performance was evaluated at the level of individual raters and at the group level (experienced vs. inexperienced). Analyses included sensitivity, specificity, accuracy, and balanced accuracy (BA), defined as (sensitivity + specificity)/2, with histological diagnosis serving as the gold standard. Patient-level decisions were derived by majority vote (≥4 out of 6 raters); ties (3–3) were classified as benign. Interrater agreement was assessed by calculating complete agreement, average error rate, and Fleiss’ κ for overall multi-rater agreement; pairwise Cohen’s κ values were additionally computed for visualization (heatmaps).

Comparisons between the two assessment approaches for paired binary outcomes (e.g., sensitivity, specificity, and accuracy at the patient level) were performed using McNemar’s test. Group comparisons between experienced and inexperienced raters were carried out using χ^2^ tests for independent proportions; Fisher’s exact test was applied when expected cell counts were <5. Proportions were calculated together with exact 95% binomial confidence intervals. Fleiss’ and Cohen’s κ coefficients were estimated with 95% confidence intervals based on standard asymptotic methods. Finally, misclassification rates were analyzed separately for benign and malignant histological entities at the patient level (majority vote, ≥4 out of 6 raters), restricted to diagnoses with at least three cases.

No formal a priori power calculation was performed. Given the cohort size and the proportion of malignant cases, the study allows reasonably precise estimates of diagnostic performance and interrater agreement at the cohort level, whereas subgroup analyses should be interpreted as exploratory. Statistical analyses were performed using Microsoft Excel for Microsoft 365 version 2408 (Microsoft Corporation, Redmond, WA, USA) and R 4.5.1 (R Foundation for Statistical Computing, Vienna, Austria).

## 3. Results

### 3.1. Patient Characteristics

A total of 146 patient cases were included in the analysis. Histological diagnoses and their distribution are shown in Table 3. The most frequent benign lesions were Reinke’s edema (22.6%), papilloma (11.6%), and polyp (8.9%). Malignant cases were dominated by squamous-cell carcinoma (13.7%), followed by carcinoma in situ (7.5%). Dysplasias accounted for 15.8% of all cases, with mild dysplasias being categorized as benign, whereas moderate and severe dysplasias as well as carcinoma in situ (CIS) were categorized as malignant for statistical analysis.

### 3.2. Diagnostic Performance of Individual Raters

Diagnostic accuracy was first assessed for each rater individually, using histopathology as the gold standard. In Assessment Approach 1 (narrow-angle PVC = malignant), sensitivities ranged from 28.2% to 64.1%, specificities from 72.0% to 93.5%, and overall accuracy from 69.2% to 82.2% (Table 4).

In Assessment Approach 2 (any PVC = malignant), sensitivities were consistently higher, ranging from 48.7% to 94.9%, while specificities decreased to 61.7–78.5%, with accuracies between 70.5% and 76.0% (Table 5).

### 3.3. Group-Level Diagnostic Performance (Experienced vs. Inexperienced)

When stratified by experience, group-level analysis showed similar accuracies between experienced and inexperienced raters in both assessment approaches. In Approach 1, inexperienced raters achieved a mean sensitivity of 39.3%, specificity of 89.4%, and accuracy of 76.0%, whereas experienced raters achieved 56.4%, 82.9%, and 75.8%, respectively (Table 6).

In Approach 2, mean sensitivity increased for both groups, but specificity decreased. Inexperienced raters achieved 75.2% sensitivity, 72.0% specificity, and 72.8% accuracy, while experienced raters reached 82.1%, 71.0%, and 74.0%, respectively (Table 7).

### 3.4. Balanced Accuracy (BA)

To further evaluate diagnostic performance, balanced accuracy was calculated as the mean of sensitivity and specificity for each rater and for the two rater groups. The results are shown in Table 8.

In Assessment Approach 1 (narrow-angle PVC = malignant), BA values ranged from 0.594 to 0.746 across all raters. The mean BA was 0.644 in the inexperienced group (R1–3) and 0.696 in the experienced group (R4–6).

In Assessment Approach 2 (any PVC = malignant), BA values were generally higher, ranging from 0.636 to 0.802. Group-level means were 0.736 for inexperienced raters and 0.766 for experienced raters.

### 3.5. Comparison of Assessment Approaches

Direct comparison between approaches revealed significant trade-offs. Sensitivity was significantly higher in Approach 2 compared to Approach 1 (*p* = 0.0018), whereas specificity was significantly lower (*p* = 0.0014). Overall accuracy did not differ significantly between the two approaches (*p* = 0.128). These trade-offs are reflected in the balanced accuracy (Table 8).

### 3.6. Subgroup Analyses

Comparisons between inexperienced (R1–3) and experienced raters (R4–6) did not reveal statistically significant differences in diagnostic accuracy (Approach 1: *p* = 0.967; Approach 2: *p* = 0.632). Similarly, no significant differences were found for sensitivity (Approach 1: *p* = 0.140; Approach 2: *p* = 0.669) or specificity (Approach 1: *p* = 0.363; Approach 2: *p* = 0.878).

### 3.7. Interrater Agreement

Interrater agreement among the six raters is summarized in Table 9. In Assessment Approach 1 (narrow-angle PVC = malignant), complete agreement across all raters was observed in 53.4% of cases, with an average error rate of 24.1%. Fleiss’ κ value was 0.367, indicating only fair agreement.

In Assessment Approach 2 (any PVC = malignant), complete agreement increased to 64.4% of cases, with a slightly higher average error rate of 26.6%. Fleiss’ κ improved substantially to 0.687, corresponding to substantial agreement.

**Figure 1:**
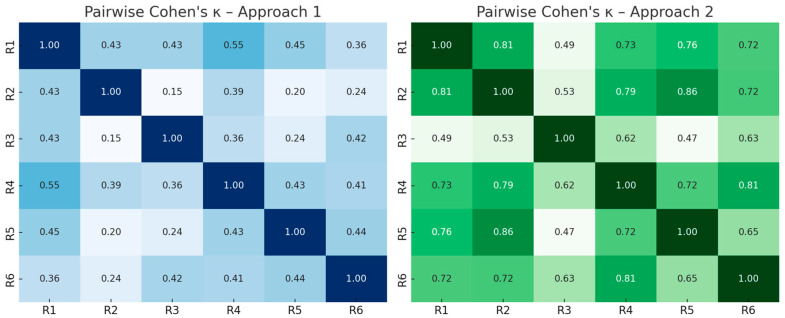
Heatmaps of pairwise Cohen’s κ values between all six raters in Approach 1 (**left**) and Approach 2 (**right**).

To visualize agreement patterns between individual raters, pairwise Cohen’s κ values were computed and are displayed as heatmaps in Figure 1. The graphical overview highlights that agreement was lower and more heterogeneous in Approach 1, with κ values mostly between 0.2 and 0.5. In contrast, Approach 2 yielded consistently higher agreement across rater pairs, with multiple κ values exceeding 0.6, indicating substantially improved reliability. Notably, experienced raters (R4–6) demonstrated higher internal agreement compared to inexperienced raters (R1–3).

### 3.8. Analysis of Misclassification Rates

Error rates were analyzed separately for benign and malignant histological entities at the patient level using majority vote, restricted to diagnoses with at least three cases (Table 10).

In Assessment Approach 1 (narrow-angle PVC = malignant), frequent false positives were observed in papillomas (41%), hyperkeratosis (33%), and mild dysplasia (27%). False negatives occurred particularly in squamous-cell carcinoma (50%), carcinoma in situ (36%), and severe dysplasia (33%).

In Assessment Approach 2 (any PVC = malignant), papillomas were consistently misclassified as malignant (100%). Hyperkeratosis (56%) and mild dysplasia (33%) also showed relevant false-positive rates. By contrast, false negatives decreased substantially: only 20% of squamous-cell carcinomas were misclassified, and all CIS and severe dysplasias were correctly classified at the patient level.

## 4. Discussion

### 4.1. Clinical Importance of Superficial Vascular Changes

The evaluation of vascular patterns in vocal fold lesions has become an indispensable part of modern laryngological diagnostics. Tumor-driven angiogenesis induces structural alterations of the superficial microvasculature in the lamina propria, most prominently the formation of intrapapillary capillary loops (IPCLs), which can be visualized endoscopically [17,18]. Historically, Kleinsasser (1962), through the introduction of microlaryngoscopy, already emphasized the diagnostic relevance of pathological vascular patterns for early detection of carcinoma [19]. He described “hook-shaped capillaries, corkscrew and hairpin capillaries, as well as irregular, dilated, and fragile vessels” as features observed exclusively in precancerous and malignant lesions [19]. These observations laid the foundation for the modern concept of vascular changes as diagnostic indicators of malignant transformation.

The technical progress of endoscopy, including the introduction of high-definition and 4K video systems, as well as image-enhanced endoscopy (IEE) methods such as narrow-band imaging (NBI), has significantly improved the ability to assess vascular changes. Although white-light (WL) endoscopy is the routine standard in clinical practice, its ability to reliably detect early or subtle malignant changes is inferior compared to image-enhanced modalities [1,2]. NBI improves visualization of capillary patterns by enhancing hemoglobin contrast and has been shown to outperform WL alone. Kraft et al. reported a sensitivity of 97% for WL + NBI versus 79% for WL alone, with comparable specificity (96% vs. 95%) in detecting laryngeal carcinoma and precursor lesions [20]. In a large prospective study including 279 patients, Piazza et al. confirmed that NBI achieved a sensitivity of 97% versus 80% for WL, again with comparable specificity, thereby establishing NBI as the superior modality for the assessment of laryngeal carcinoma [6]. Meta-analyses further support this conclusion: Sanda et al. pooled results from 17 studies and reported a sensitivity of 0.87 and specificity of 0.90 for NBI in detecting malignant laryngeal lesions [4], while Saraniti et al. demonstrated that NBI consistently outperforms WL across both preoperative and intraoperative settings [3]. Ahmadzada et al. also confirmed the diagnostic superiority of NBI in the evaluation of leukoplakia, with pooled sensitivity and specificity of 0.93 and 0.82, respectively [21].

### 4.2. Role of Enhanced Contact Endoscopy (ECE)

In overview endoscopy, the assessment of vascular changes cannot be isolated from the macroscopic appearance of the vocal fold lesion itself. Thus, the diagnostic value of vascular morphology alone is difficult to evaluate since raters inevitably take both surface characteristics and vascular patterns into account. Enhanced contact endoscopy (ECE) represents the combination of classic contact endoscopy with an image-enhanced endoscopy (IEE) modality. In contrast to the historical form of CE, which was usually performed with vital staining of the mucosa, modern ECE relies on unstained CE combined with IEE. Among these approaches, NBI-CE has emerged as the most widely applied and best-studied variant [10,11,12]. Nevertheless, other IEE technologies such as IMAGE1 S™ have also been successfully applied in combination with CE [9]. This technique allows the vascular morphology itself to be isolated and studied under direct mucosal contact at 60–150× magnification. It provides superior contrast and magnification, enabling detailed visualization of even the smallest vascular structures. Additionally, NBI has been shown to mitigate the so-called “umbrella effect”, where leukoplakic hyperkeratosis obscures the underlying vasculature [22]. ECE further enhances detection of microvascular changes at the lesion margins by directly exposing the subsurface vessels—even those masked in overview modes—improving diagnostic accuracy in leukoplakia and dysplasia. By minimizing the influence of gross morphology and the umbrella effect, ECE isolates vascular architecture at 60–150× contact magnification, enabling fine-grained assessment of IPCL patterns. Beyond these technical advantages, the clinical utility of ECE ultimately depends on how vascular morphologies are classified. Within this framework, perpendicular vascular changes (PVC) have emerged as the most relevant diagnostic feature [10,11,13].

### 4.3. Diagnostic Trade-Offs of Perpendicular Vascular Changes

The classification of vascular changes aims to improve diagnostic precision in the assessment of vocal fold lesions. Within the European Laryngological Society (ELS) guideline, perpendicular vascular changes (PVC) are considered a key indicator of malignancy, while longitudinal vascular changes (LVC) are more commonly associated with benign lesions [10]. In their original proposal, Arens et al. also introduced the subclassification of PVC into narrow-angle (naPVC) and wide-angle (waPVC) intrapapillary capillary loops (IPCLs). This refinement was intended to address one of the major pitfalls in endoscopic vascular diagnostics—namely, that papillomas also show PVC, potentially mimicking malignancy. Subsequent work on overview endoscopy confirmed this diagnostic separation: Šifrer et al. (2020) reported naPVC in 96.2% of malignant lesions, whereas waPVC predominated in 80% of papillomas [14].

In our study, the two diagnostic approaches mirrored these conceptual differences: using any PVC as malignancy criterion resulted in higher sensitivity but lower specificity, while restricting the criterion to naPVC improved specificity at the expense of sensitivity. Our misclassification analysis (Table 10) quantifies these trade-offs by diagnosis: papilloma dominated false-positive assignments under the “any PVC” approach, whereas squamous-cell carcinoma and CIS accounted for most false negatives when restricting the criterion to naPVC. This diagnostic trade-off reflects the clinical dilemma between minimizing false negatives and avoiding false positives. A previous study from our group (Davaris et al., 2020 [11]), which analyzed a smaller cohort of 68 patients, also reported high sensitivity (95.5%) but only moderate specificity (63.0%) for PVC as a malignancy marker in NBI-based contact endoscopy (NBI-CE). Importantly, that analysis treated PVC only as a dichotomous feature (present vs. absent), without distinguishing between narrow- and wide-angled loops [11]. Schöninger et al. (2021) likewise confirmed PVC as a strong malignancy indicator using ECE, although without applying the na/waPVC distinction [23].

Additional studies have supported the clinical value of the ELS classification. Missale et al. (2021) [24] demonstrated robust diagnostic performance of the ELS vascular criteria in a large multicenter cohort of laryngeal lesions, and Yildirim et al. (2021) [9] showed that the system can be applied not only with NBI but also with other image-enhanced endoscopy modalities such as IMAGE1 S™. These findings underscore the versatility of the ELS system across different technologies [9,24].

By contrast, many earlier NBI studies in overview endoscopy—including those by Piazza (2010) and Bertino (2015)—relied on the five-tier Ni classification [6,7,25]. While these studies reported high sensitivity for detecting malignant lesions, their results are not directly comparable to PVC-based approaches, since vascular and macroscopic lesion features were evaluated together. As Kántor et al. (2022) emphasized, the Ni classification is more complex and less reproducible, whereas the simplified dichotomous PVC framework of the ELS guideline is easier to apply in clinical practice and more intuitive for training purposes [2].

Taken together, current evidence highlights the diagnostic value of PVC within the ELS system, but also the limitations inherent to different assessment approaches. Considering all PVC as malignant increases sensitivity but carries a risk of false positives, especially in papillomas, whereas restricting the criterion to naPVC yields higher specificity but reduces sensitivity. In clinical routine, the combination of vascular assessment with macroscopic lesion characteristics—as is the case in overview endoscopy—can achieve very high diagnostic accuracy. However, when vascular morphology is assessed in isolation, as in ECE, further refinement of the ELS classification will likely be required to optimize diagnostic performance.

Clinically, these trade-offs highlight the importance of context-specific decision-making: in papilloma-prevalent settings, naPVC improves specificity, while in oncologic surveillance, considering any PVC maximizes sensitivity for high-grade lesions. While these findings highlight the diagnostic potential of PVC within the ELS system, their clinical value ultimately depends not only on sensitivity and specificity but also on the consistency with which different raters can apply these criteria. The reproducibility of vascular classification is therefore a key prerequisite for its reliable use in daily practice.

### 4.4. Reproducibility of PVC Assessment

A major prerequisite for integrating vascular classification systems into clinical routine is their reproducibility across raters with different levels of expertise. In our study, experienced raters consistently achieved higher agreement and diagnostic accuracy than inexperienced raters, confirming that familiarity with vascular morphology influences diagnostic reliability. The κ-values were significantly higher in the experienced group, accompanied by superior sensitivity and specificity, whereas inexperienced raters tended to overestimate malignancy in cases with ambiguous vascular changes. This was reflected in our data, where experienced raters achieved higher κ values and a more favorable balance of sensitivity and specificity than inexperienced raters.

These findings are in line with previous reports using enhanced contact endoscopy. Davaris et al. (2020) observed substantial overall agreement for PVC assessment, with κ-values reaching almost perfect levels among experienced otolaryngologists, but only moderate values among less experienced raters [11]. Schöninger et al. (2021) also found that ECE improved interrater reliability compared to white-light and standard NBI overview endoscopy, underlining the advantages of high magnification and contrast. ECE’s high magnification and contrast likely explain the improved agreement compared with overview WL/NBI endoscopy [23].

Several other studies have addressed the reproducibility of vascular classifications. Mehlum et al. (2020) compared different systems in ECE and reported the best κ-values for the ELS classification, significantly higher than those achieved with the Ni or Puxeddu classifications [13]. Similarly, Missale et al. (2021) validated the ELS guideline in a large multicenter cohort, demonstrating very high interobserver agreement [24]. By contrast, studies based on the Ni classification often report only moderate κ-values, reflecting the greater complexity and subjectivity of a five-tiered system [2]. Our reproducibility findings with high-magnification NBI-CE align with prior overview NBI work showing improved inter- and intraobserver agreement versus WL [8], suggesting that vessel-contrast enhancement benefits consistency across experience levels. In routine care, the binary PVC construct is typically faster to teach and apply than multi-tiered scales, which may further support its adoption for triage and training. Despite these strengths, certain lesion types remain diagnostically challenging even under ECE, as highlighted by our misclassification patterns.

Taken together, these results suggest that the ELS classification provides a relatively robust framework for classifying vascular changes, but its reproducibility is still influenced by rater experience and the chosen assessment approach. In particular, differentiating between narrow- and wide-angled PVC remains challenging even for experts, and represents a potential source of variability. In our cohort, this translated into a substantial improvement of interrater agreement from fair (κ ≈ 0.37) to substantial (κ ≈ 0.69) when shifting from naPVC-only to any PVC criteria. Simplifying the decision to the presence or absence of PVC improves agreement, but at the cost of diagnostic specificity. Future refinements of the ELS classification may therefore need to incorporate clearer morphological descriptors or quantitative, automated tools to minimize interrater variability.

Similar challenges of observer dependence and interrater variability have been described for other laryngeal imaging modalities. Laryngeal ultrasonography is likewise highly operator dependent and subject to differences in image acquisition and interpretation. In their review, Cergan et al. emphasized that standardized examination protocols and systematic training are crucial prerequisites for reliable ultrasonographic assessment of the larynx [26]. This parallels our findings for NBI-CE, where both rater expertise and the chosen vascular classification strategy substantially influenced diagnostic performance and reproducibility. Together, these observations suggest that improving interobserver agreement is a general challenge across laryngeal imaging techniques and not limited to vascular assessment with NBI-CE.

While reproducibility is a prerequisite for clinical applicability, even high agreement does not fully resolve diagnostic pitfalls. Certain lesion types remain particularly challenging in CE-NBI, as discussed in the following section.

### 4.5. Diagnostic Pitfalls in PVC-Based Assessment

Despite the overall diagnostic value of PVC, certain lesion types continue to present major challenges. In our study, papillomas emerged as the most frequent source of false-positive classifications when all PVC were used as a malignancy criterion. This reflects the fact that papillomas, although benign, exhibit prominent perpendicular vascular patterns that mimic those of malignant lesions. Narrow-angle PVC provide greater specificity, but their reliable recognition requires experience and is not always feasible in practice.

False negatives were mainly observed in squamous-cell carcinomas and dysplastic lesions, especially when vascular changes were subtle or combined with longitudinal patterns. It is also conceivable that PVC remained undetected during ECE, either because of technical limitations in magnification and focus or because representative frames were not selected for evaluation. Such factors may have contributed to cases where histology confirmed malignancy but vascular changes were missed by raters.

False positives, on the other hand, may partly reflect the biological overlap between papillomas and carcinomas, but in some cases could also be due to sampling error. If the biopsy was not fully representative of the lesion, histology—the diagnostic gold standard used for calculating performance metrics—might have underestimated malignant potential.

Beyond these technical and procedural issues, additional morphological features such as the homogeneity, density, and symmetry of PVC are not explicitly captured by the current ELS classification. These finer vascular characteristics could have diagnostic value, but remain underexplored and may explain part of the misclassification observed in both our data and previous studies.

An earlier study from our group (Davaris et al., 2020) also reported frequent misclassification of papillomas as malignant when PVC were applied as the sole diagnostic marker in NBI-ECE [11]. Schöninger et al. (2021) similarly described errors in both papillomas and dysplastic lesions, underlining the overlap in vascular morphology [23]. Šifrer et al. (2020) also highlighted the role of naPVC in differentiating benign from malignant lesions [14]. However, it is important to note that some of the studies were based on overview endoscopy, where both vascular and gross morphological features of the lesion were visible. This makes their findings less directly comparable to studies such as ours, which relied exclusively on vascular morphology assessed with enhanced contact endoscopy.

Another persistent challenge lies in the interpretation of dysplastic lesions. While high-grade dysplasia and carcinoma in situ often demonstrate naPVC, early or low-grade dysplasia may present with ambiguous vascular changes. This variability complicates classification and may explain the lower κ-values observed for premalignant and malignant categories compared to benign lesions.

### 4.6. Limitations

This study has several limitations. It is single-center and retrospective, with a moderate overall sample size and limited numbers in some diagnostic subgroups (e.g., severe dysplasia, rare benign lesions). Still images were manually extracted from video recordings, which may introduce selection bias and do not fully capture intra-case variability. Furthermore, patients for whom no set of diagnostically usable NBI-CE images could be obtained were excluded during data curation. Although this affected fewer than 5% of otherwise eligible cases, such preselection by image quality may slightly overestimate diagnostic performance and limit generalizability to unselected real-world cohorts. Moreover, during the primary intraoperative examination with NBI-CE, clinically relevant regions with vascular alterations may in rare cases have been overlooked, meaning that such features would not be represented in the extracted dataset. Histopathology served as the diagnostic gold standard, yet the representativeness of biopsy or excisional specimens cannot be guaranteed in all cases, potentially leading to sampling error. Device-specific factors and the pre-specified majority-vote rule (≥4/6) could bias patient-level outcomes. In addition, each case was rated only once per observer, so intra-rater reproducibility could not be assessed. These aspects reduce generalizability and highlight the need for multicenter validation. Despite these methodological limitations, our findings provide a valuable basis for future developments. One promising avenue is the integration of artificial intelligence (AI), which may help to address some of the observed diagnostic shortcomings.

### 4.7. Role of Artificial Intelligence and Future Directions

The diagnostic pitfalls described above illustrate the limitations of human-based vascular assessment and have stimulated increasing interest in artificial intelligence (AI). Recent advances demonstrate deep learning’s potential: Esmaeili et al. (2019) showed that automated vascular pattern characterization in contact endoscopy outperformed manual evaluation by otolaryngologists [27]. Xu et al. (2023) achieved excellent diagnostic accuracy using a Densenet201 model trained on laryngoscopic images, while He et al. (2021) further extended these approaches to histopathological datasets [28,29]. Azam et al. (2022) applied a YOLO-based algorithm for real-time detection of laryngeal carcinoma on both WL and NBI videolaryngoscopy, demonstrating the feasibility of live AI-assisted endoscopy [30]. In parallel, Paderno et al. (2022) explored “videomics”, using convolutional neural networks for automated classification of laryngeal lesions during endoscopic imaging, further underlining the translational potential of AI in this field [31].

Nevertheless, AI systems do not resolve the biological overlap between papillomas, dysplasias, and carcinomas. Most algorithms are trained to reproduce human classification, thereby perpetuating existing weaknesses, and their “black box” nature limits pathophysiological insight.

Future research should therefore aim to combine AI with refined classification schemes and quantitative vascular descriptors such as vessel density, diameter variability, and branching complexity. Larger multicenter datasets will be essential to ensure generalizability. Ultimately, combining AI tools with optimized classification schemes—supported by prospective multicenter trials—could establish vascular assessment via ECE as a robust, objective tool in everyday laryngology.

## 5. Conclusions

This study confirms the central diagnostic role of perpendicular vascular changes (PVC) in the endoscopic assessment of vocal fold lesions using NBI-CE. While PVC reliably indicate malignant potential, their interpretation is influenced by the chosen assessment strategy—considering all PVC improves sensitivity but reduces specificity, whereas restricting diagnosis to narrow-angle PVC achieves the opposite. Reproducibility was higher among experienced raters, but interrater agreement was overall superior when PVC were scored without further subclassification. Papillomas and dysplastic lesions remain the most challenging differential diagnoses, and pitfalls in their evaluation highlight the need for refined vascular descriptors and standardized image selection.

Future improvements will depend on both advances in classification systems and the integration of artificial intelligence to support objective image interpretation. Broader clinical adoption of ECE, alongside multicenter validation, will be crucial for establishing vascular assessment as a reliable tool in daily laryngological practice.

## Figures and Tables

**Table 1:** Evaluation criteria for classification of patient cases based on NBI-CE images.

Category	Definition
LVC	Presence of longitudinal vascular changes only, according to ELS classification
Narrow-angle PVC	Predominantly narrow-angle PVC, according to ELS classification (regardless of LVC presence)
Wide-angle PVC	Predominantly wide-angle PVC, according to ELS classification (regardless of LVC presence)

**Table 2:** Assessment approaches for classification of patient cases according to ELS criteria.

Assessment Approach	Malignancy Criterion
1	Narrow-angle PVC present
2	Any PVC present (narrow- or wide-angle)

**Table 3:** Histological diagnoses of all cases (*n* = 146).

Diagnosis	*n*	%
Reinke’s edema	33	22.6
Papilloma	17	11.6
Polyp	13	8.9
Hyperkeratosis	9	6.2
Cyst	7	4.8
Hyperplasia	4	2.7
Amyloidosis	3	2.1
Granuloma	2	1.4
Hemangioma	2	1.4
Inflammation	1	0.7
Nodule	1	0.7
Mild dysplasia (counted as benign)	15	10.3
*Benign total*	*107*	*73.3*
Moderate dysplasia	2	1.4
Severe dysplasia	6	4.1
Carcinoma in situ	11	7.5
Squamous-cell carcinoma	20	13.7
*Malignant total*	*39*	*26.7*
**Total**	**146**	**100.0**

Values are number of cases and percentages. Italic rows indicate benign and malignant subtotals; the bold row indicates the overall total.

**Table 4:** Diagnostic performance of individual raters using Assessment Approach 1 (narrow-angle PVC = malignant).

Rater	Sensitivity	Specificity	Accuracy
Rater 1 (inexperienced)	0.385	0.841	0.719
Rater 2 (inexperienced)	0.513	0.935	0.822
Rater 3 (inexperienced)	0.282	0.907	0.740
Rater 4 (experienced)	0.641	0.850	0.795
Rater 5 (experienced)	0.615	0.720	0.692
Rater 6 (experienced)	0.436	0.916	0.788

**Table 5:** Diagnostic performance of individual raters using Assessment Approach 2 (any PVC = malignant).

Rater	Sensitivity	Specificity	Accuracy
Rater 1 (inexperienced)	0.846	0.692	0.733
Rater 2 (inexperienced)	0.923	0.682	0.747
Rater 3 (inexperienced)	0.487	0.785	0.705
Rater 4 (experienced)	0.769	0.748	0.753
Rater 5 (experienced)	0.949	0.617	0.705
Rater 6 (experienced)	0.744	0.766	0.760

**Table 6:** Group-level diagnostic performance in Assessment Approach 1 (narrow-angle PVC = malignant).

Group	Sensitivity	Specificity	Accuracy
Inexperienced (R1–3)	0.393	0.894	0.760
Experienced (R4–6)	0.564	0.829	0.758

**Table 7:** Group-level diagnostic performance in Assessment Approach 2 (any PVC = malignant).

Group	Sensitivity	Specificity	Accuracy
Inexperienced (R1–3)	0.752	0.720	0.728
Experienced (R4–6)	0.821	0.710	0.740

**Table 8:** Balanced Accuracy (BA) of individual raters and groups in Assessment Approach 1 (narrow-angle PVC = malignant) and Assessment Approach 2 (any PVC = malignant).

Rater/Group	Approach 1 (Narrow-Angle PVC)	Approach 2 (Any PVC)
Rater 1 (inexperienced)	0.613	0.769
Rater 2 (inexperienced)	0.724	0.802
Rater 3 (inexperienced)	0.594	0.636
Rater 4 (experienced)	0.746	0.758
Rater 5 (experienced)	0.668	0.783
Rater 6 (experienced)	0.676	0.755
**Inexperienced (R1–3)**	**0.644**	**0.736**
**Experienced (R4–6)**	**0.696**	**0.766**

Bold rows represent group averages for inexperienced (R1–3) and experienced (R4–6) raters.

**Table 9:** Interrater agreement in both assessment approaches (*n* = 146).

Metric	Approach 1 (Narrow-Angle PVC)	Approach 2 (Any PVC)
Complete agreement (%)	53.4	64.4
Average error rate (%)	24.1	26.6
Fleiss’ κ	0.367	0.687

**Table 10:** Patient-level misclassification rates by diagnosis (majority vote, diagnoses ≥ 3 cases).

Diagnosis	*n*	Approach 1	Approach 2
Papilloma	17	7/17 (41%) FP	17/17 (100%) FP
Hyperkeratosis	9	3/9 (33%) FP	5/9 (56%) FP
Mild dysplasia	15	4/15 (27%) FP	5/15 (33%) FP
Severe dysplasia	6	2/6 (33%) FN	0/6 (0%) FN
Carcinoma in situ	11	4/11 (36%) FN	0/11 (0%) FN
Squamous-cell carcinoma	20	10/20 (50%) FN	4/20 (20%) FN

## Data Availability

The NBI-CE still images and labels analyzed in this study are part of the publicly available CE-NBI dataset: Zenodo (doi:10.5281/zenodo.6674034) and Scientific Data (doi:10.1038/s41597-023-02629-7). No new primary data were generated. Derived analysis outputs are available from the corresponding author on reasonable request.

## Supplementary Materials

The following supporting information can be downloaded at: https://www.mdpi.com/article/10.3390/diagnostics15233051/s1, Video S1: Example of longitudinal vascular changes (LVC) in a Reinke’s edema. Video S2: Example of perpendicular vascular changes (PVC) in vocal fold papilloma.

## Abbreviations

The following abbreviations are used in this manuscript:

AI	artificial intelligence
BA	balanced accuracy
CE	contact endoscopy
CE-NBI	Contact Endoscopy–Narrow-Band Imaging (dataset)
CIS	carcinoma in situ
DCNN	deep convolutional neural network
ECE	enhanced contact endoscopy (CE + IEE)
ELS	European Laryngological Society
FN	false negative
FP	false positive
FPR	false-positive rate
IEE	image-enhanced endoscopy
IPCL	intrapapillary capillary loop
LVC	longitudinal vascular changes
NBI	narrow-band imaging
NBI-CE	narrow-band imaging–assisted contact endoscopy
naPVC	narrow-angle perpendicular vascular changes
PVC	perpendicular vascular changes
SCC	squamous-cell carcinoma
TPR	true-positive rate
waPVC	wide-angle perpendicular vascular changes
WHO	World Health Organization
WL	white light
